# Histone-Mutant Glioma: Molecular Mechanisms, Preclinical Models, and Implications for Therapy

**DOI:** 10.3390/ijms21197193

**Published:** 2020-09-29

**Authors:** Maya S. Graham, Ingo K. Mellinghoff

**Affiliations:** 1 Department of Neurology, Memorial Sloan Kettering Cancer Center, New York, NY 10065, USA; grahamm3@mskcc.org; 2 Human Oncology and Pathogenesis Program, Memorial Sloan Kettering Cancer Center, New York, NY 10065, USA

**Keywords:** pediatric high-grade glioma, diffuse midline glioma, oncohistone, H3K27M

## Abstract

Pediatric high-grade glioma (pHGG) is the leading cause of cancer death in children. Despite histologic similarities, it has recently become apparent that this disease is molecularly distinct from its adult counterpart. Specific hallmark oncogenic histone mutations within pediatric malignant gliomas divide these tumors into subgroups with different neuroanatomic and chronologic predilections. In this review, we will summarize the characteristic molecular alterations of pediatric high-grade gliomas, with a focus on how preclinical models of these alterations have furthered our understanding of their oncogenicity as well as their potential impact on developing targeted therapies for this devastating disease.

## 1. Introduction

Pediatric high-grade glioma (pHGG) is the leading cause of cancer death in children, with a median overall survival of less than one year [1]. This dismal prognosis has remained stagnant for decades despite remarkable progress in other tumor types. The more recent exponential increase in our ability to characterize human tumor biopsies at a molecular level has spurred further investigation of the biological underpinnings of this disease. 

While adult high-grade glioma is typified by a combination of mutations and gene copy alterations in core signaling pathways, epigenetic dysregulation has emerged as a prominent feature in pediatric tumors [2,3]. The field of cancer epigenetics is expanding, and mutations in chromatin regulators such as readers, writers, and erasers of histone modifications have now been catalogued in a variety of different human cancers [4]. However, the first cancer-associated mutations in histone genes themselves—so-called “oncohistones”—were discovered in pHGG. Two groups simultaneously described mutually exclusive recurrent somatic missense mutations in the amino tail of histone H3 genes: a lysine-to-methionine substitution at position 27 of histone 3.1 or 3.3 (H3K27M) and a glycine-to-arginine (or valine) substitution at position 34 of histone 3.3 (H3.3G34R/V) [5,6]. Subsequent characterization of a larger number of tumors revealed that oncohistones delineate subgroups of pHGG with distinct ages of onset, anatomical locations, and coincident mutations [7]. H3K27M mutations are found in the vast majority of midline pHGG, including over 80% of those in the pons (previously known as diffuse intrinsic pontine glioma or DIPG) and 60% of non-brainstem midline structures such as thalamus [8] and spinal cord [9]. By contrast, H3.3G34R/V mutations are found in 16–20% of supratentorial pHGG of the cerebral hemispheres and are exemplified by an older age of onset [9,10]. H3K27M mutations have come to be seen as a defining characteristic of midline pHGG, as underscored by their inclusion as a new molecularly-based diagnostic subgroup termed diffuse midline glioma (DMG) in the 2016 update of the World Health Organization (WHO) criteria [11]. Despite their combined grouping in this diagnostic classification, H3.3K27M tumors and H3.1K27M tumors do seem to typify distinct clinical categories, with H3.1K27M tumors restricted almost exclusively to the pons and demonstrating a slightly younger age of onset [8]. 

**Figure 1:**
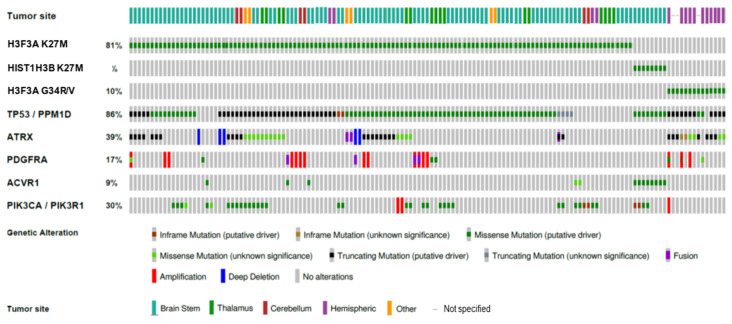
Oncoprint depicting tumor locations and selected typical genetic alterations in histone-mutant pHGG. Original data publicly available in PedcBioPortal.

In addition to the distinct clinical presentation, tumors with specific histone mutations have a distinct set of cooccurring genetic alterations that appear to contribute to their oncogenicity. For example, H3.3K27M mutant pHGG are enriched for *PDGFRA* amplification, while H3.1K27M tumors are associated with *ACVR1* mutations [10,12,13]. H3.3G34R/V tumors, meanwhile, are enriched for mutations in the histone H3.3 chaperone complex protein *ATRX* [5,9,10]. *TP53* mutation is common in all histone-mutant glioma subsets (Figure 1). Serial tumor biopsies, autopsy sampling, and clonal evolution analyses suggest that oncohistones (and their obligate partner mutations) remain detectable throughout the course of disease, with oncohistone mutation as an initial event followed closely by p53 pathway alteration and, subsequently, subclonal growth factor signaling mutation [14,15]. 

Despite this comprehensive characterization of pediatric histone-mutant glioma, clinical mysteries remain. Oncohistone mutation occurs in adult patients as well, albeit much more rarely. Small case series suggest that H3K27M continues to be found primarily in midline tumors in adults, though with a shift in predilection toward thalamic and spinal cord involvement [16,17,18], and that H3G34R/V continues to be found primarily in cortical tumors [19]. However, our understanding of their clinical trajectory, as well as whether they harbor the same obligate partner mutations as their pediatric counterparts, remains to be determined. In aggregate, the discovery of driver oncohistone mutations has led to an explosion of research into their molecular consequences, with the hope that defining these consequences will ultimately lead to improved targeted therapies.

## 2. Experimental Models

Prior to the discovery of the molecular hallmarks described above, preclinical models of pHGG were restricted to patient-derived xenografts, which were in and of themselves challenging to procure given the often-precarious neuroanatomical location of these tumors. They are also limited in their ability to serve as preclinical subjects for immunotherapy studies given the need for immunocompromised host mice. The subsequent advances in our understanding of pHGG biology have allowed for the development of increasingly faithful and diverse preclinical models. Several groups have capitalized on the elucidation of defining concurrent mutations by establishing genetically engineered mouse models (GEMMs), either by conditional genetic manipulation or xenografts of engineered transformed cells. In keeping with the relative abundance of research on H3.3K27M molecular mechanisms, the majority of preclinical models to date have focused on this particular oncohistone [20,21] (Table 1). Interestingly, H3.3K27M as an isolated driver has failed to be oncogenic in multiple models [22,23,24], perhaps in keeping with a predominant effect of “setting the stage” for subsequent oncogenic insults in a uniquely vulnerable cell-of-origin. Recent elaboration of H3.1K27M-specific partner mutations and molecular effects has allowed for the successful development of models for this oncohistone as well. There are no published preclinical models of H3.3G34R/V cortical tumors as of yet, though preliminary data have been presented [25].

### 2.1. Ex Vivo Somatically Altered NPCs

Genetic hallmarks of pHGG have been leveraged in the development of xenograft models using engineered cells. The first such model used lentiviral transduction of H3.3K27M, activated PDGFRA^D842V^, and shRNA targeting p53 in neural progenitor cells (NPCs) derived from human embryonic stem cells, which were then orthotopically transplanted into early postnatal immunocompromised mice [26]. The resultant tumors diffusely infiltrated the pons within months and showed elevated proliferation consistent with malignant glioma (Ki67 10%), though they did not show the necrosis or microvascular proliferation that are pathognomonic for glioblastoma. By contrast, tumors failed to form when H3.3 wild-type (WT) was transduced in place of the K27M mutant. 

An analogous approach was used to combine H3.3K27M with PDGF-B overexpression in embryonic mouse forebrain NPCs, which were then transplanted into the pons of adolescent immunodeficient mice [27]. This led to accelerated formation of pontine HGG as compared with H3.3WT. Notably, the introduction of H3.3K27M in p53-knockout NPCs did not result in tumor formation, contrary to findings in an in utero electroporation model described below [24]. The combination of all three hits (H3.3K27M, p53 loss, and activated PDGFR signaling) was not assessed in this system.

### 2.2. RCAS/tv-a System

The first GEMM of DMG predates the discovery of oncohistones and utilized the RCAS/tv-a gene delivery system, which targets ectopic expression of the avian virus receptor tv-a to specific cells, thus allowing for selected expression of oncogenes delivered via injection of avian-based RCAS virus-producing cells [42]. Becher and colleagues successfully generated brainstem high-grade gliomas by targeting PDGF-B combined with Cre-mediated p16^INK4A/ARF^ loss to nestin-positive neural progenitors around the fourth ventricle in neonatal mice [28]. 

This model was modified once H3K27M oncohistones were found, using RCAS vectors for PDGF-B, H3.3K27M, and Cre-mediated p53 loss in the same hindbrain nestin-positive progenitors [29]. The combination of all three genetic insults was required for pHGG formation, though H3.3K27M + p53 loss was sufficient to produce proliferating clusters of cells that were not seen in H3.3WT mice. Further analysis of this model confirmed that high-grade tumors were only formed in the presence of H3.3K27M mutant (and not WT) virus, which also decreased tumor latency [22]. Targeting of these RCAS viruses to Pax3-positive brainstem cells was also capable of driving glioma development, though notably there was no difference in tumor incidence, latency, or grade between H3.3K27M and H3.3WT mice [30]. 

More recently, the RCAS/tv-a system has been successfully applied to H3.1K27M, with targeting of H3.1K27M, multiple clinically relevant ACVR1 mutants and Cre-mediated p53 loss in nestin-positive brainstem progenitors [31]. While the combination of these three genetic hits resulted in premalignant glioma-like lesions, they were insufficient for frank tumor formation despite multiple different combinations and the addition of pten loss. Brainstem gliomas formed only when PDGF-A was added as a fourth insult; in this setting, the presence of combined H3.1K27M and mutant ACVR1 served to increase tumor incidence and grade while decreasing tumor latency. It should be noted that mutations or amplifications activating PDGF signaling are relatively rare in the subset of H3.1K27M + ACVR1 mutant pHGG [10], though it is possible that PDGF signaling is indirectly activated in these tumors by other means.

### 2.3. In Utero Electroporation (IUE)

While the above models recapitulated many of the histological and molecular features of DMG, they were not entirely faithful to the relevant genetic alterations of this tumor as they utilize exogenous expression of PDGF ligand as opposed to cell-intrinsic activation of PDGF receptor. This is largely due to the restricted size of transcripts amenable to RCAS incorporation. As such, these models may erroneously incorporate paracrine effects on cells other than the putative cell-of-origin and will fail to capture any non-canonical effects that may be mediated by mutant receptors. 

Preclinical models incorporating PDGF receptor perturbation have been established harnessing in utero electroporation of oncogenic transposon vectors injected into the ventricles of the developing mouse, allowing genomic incorporation of the transposon only in subventricular neural progenitor cells [43,44]. Strikingly, no tumors formed when combining H3.3K27M and p53 loss driven by either nestin or GFAP promoters, nor by electroporation of Sleeping Beauty-based transposons in the neonatal mouse [24]. 

In contrast, introduction of H3.3K27M via a piggyBac transposon system combined with CRISPR/Cas9-mediated loss of p53 in the embryonic mouse brain was sufficient to cause tumorigenesis with 100% penetrance in both forebrain and hindbrain. Notably, no tumors formed with the introduction of H3.3WT or H3.3G34R in this system. This was the first GEMM to show that H3.3K27M and p53 loss alone were competent to drive pHGG formation, possibly due to the embryonic (as opposed to postnatal) cell of mutation. Whether this indicates an embryonic cell-of-origin remains unsettled, as prior work in adult gliomas suggests the possibility of distinct cell of mutation and cell-of-origin populations [45]. Further fine-tuning of this model with the addition of ATRX loss and PDGFRA overexpression significantly shortened tumor latency. 

Other IUE-based models have directly compared the effects of exogenous PDGF ligand versus receptor. The combination of piggyBac transposon-mediated embryonic IUE of H3.3K27M, p53 loss, and either PDGF-B, PDGFRA^WT^, or constitutive mutant PDGFRA^D842V^ all resulted in fully penetrant glioma formation, but with distinct differences in histological characterization [32]. Exogenous PDGF ligand led to cell-extrinsic perivascular changes that contributed to the shortest latency, while WT PDGFRA led to less aggressive tumors with longer latency as compared with PDGFRA^D842V^. 

Finally, transposon-mediated somatic alteration in neonatal mice has been explored to develop a model of H3.3G34R pHGG. In an oral presentation at a recent Society for Neuro-Oncology meeting, the combination of H3.3G34R, NRAS^V12^, and shRNAs targeting p53 and ATRX was reported to form tumors, though with increased latency when compared with H3.3WT in the same context [25]. As NRAS mutation is not classic of pHGG, it remains to be seen if the underlying mechanisms of tumorigenesis in this model faithfully recapitulate those of the corresponding human tumors. Further characterization of this and other models incorporating H3.3G34R will be essential to deepening our understanding of this particular pHGG subset.

### 2.4. Transgenic Mice

The above-mentioned GEMMs all rely on oncogene expression from exogenous promotors and dictate the geography of transformation by choosing the location of virus injection or electroporation. Recent advances in conditional germline knockin mouse models have eliminated those constraints. H3.3K27M was knocked into the endogenous H3F3A locus and combined with p53 loss and the constitutively active PDGFRA^V544ins^ mutant driven by a tamoxifen-inducible Cre recombinase in neonatal nestin-positive cells throughout the developing brain [23]. This model led to spontaneous malignant brain tumor formation, with H3.3K27M driving hindbrain specificity of tumorigenesis and PDGFRA signaling driving pHGG identity. 

A similar approach was used to knock H3.1K27M and ACVR1^G328V^ into their respective endogenous loci driven by Cre recombinase in Olig2-positive oligodendrocyte precursor cells (OPCs) [34]. These two genetic insults served to arrest glial differentiation and promote proliferation, though they were insufficient to drive tumorigenesis. The addition of endogenous PIK3CA^H1047R^ knockin gave rise to spontaneous midbrain and thalamus HGG, albeit with a protracted latency of over one year. Tumorigenesis in the absence of H3.1K27M was even more protracted, suggesting this oncohistone played a role in accelerating tumorigenesis. In contrast to other GEMMs, combination with p53 loss was not explored, though p53 mutations are common in H3.1K27M tumors, which may have contributed to the prolonged tumor latency.

### 2.5. Patient-Derived Xenografts (PDXs)

Orthotopic patient-derived xenografts consist of dissociated patient tumor cells, usually passed briefly through cell culture and then implanted stereotactically into the brainstem of an immunocompromised mouse. The first PDX model of DMG was developed by Monje and colleagues in 2011 using short term neurosphere culture of early postmortem tissue from a patient with diffuse intrinsic pontine glioma [35]. While the histone-mutant status of the tumor was not known at the time and was later revealed to be H3 wild type, this protocol for transient neurosphere culture of DMG tissue followed by xenografting has subsequently been successfully applied to multiple H3K27M-mutant tumors [36,37]. Tumor latency in these models ranges from 3 to 6 months from implantation, considerably longer than in analogous PDX models of adult glioblastoma. 

More recently, systematic characterization of pHGG PDX models from both early biopsies as well as autopsy tissue has been pursued and biobanks have been established [38,39,40]. Comparison of these models may help elucidate and distinguish tumorigenic mechanisms present at diagnosis as opposed to at terminal disease, complementing tumor evolution studies done on primary human tumors [14,46]. 

Of note, while several groups have established PDXs from cortically based pediatric tumors [38,47,48], none of these have harbored H3.3G34R/V mutations. All published PDX models of histone-mutant pHGG are too numerous to specify here; however, they have been comprehensively catalogued in a recent review [41].

## 3. Molecular Mechanisms of Oncogenicity

Since the discovery of oncohistones in pHGG, substantial effort has been devoted to uncovering the molecular mechanisms by which they promote tumor formation, often leveraging the experimental models delineated above. Even initial studies showed disparate effects of different histone H3 genes and in different cell types, highlighting the nuanced consequences of chromatin dysregulation in oncogenesis. Below, we summarize the evolution of our current understanding of the downstream ramifications of oncohistone mutation in pediatric malignant glioma [49,50,51].

### 3.1. H3.3K27M

**Figure 2:**
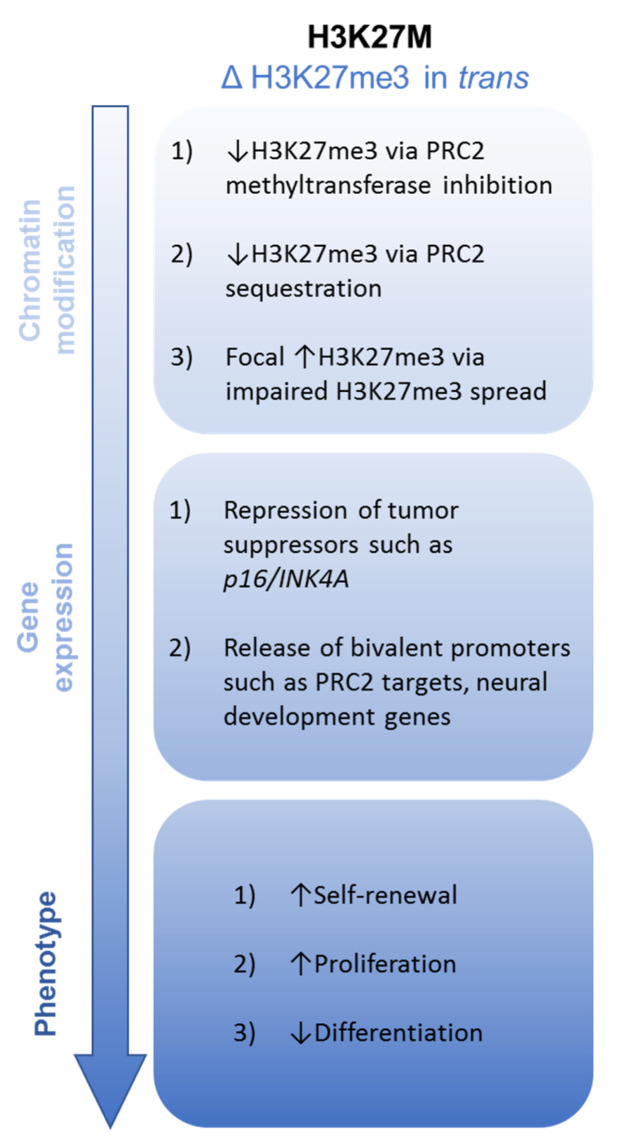
Putative oncogenic mechanisms of H3K27M in pHGG.

The consistent presence of a heterozygous K27M mutation in an individual histone H3 gene (of which there are 16 total in humans) suggests a dominant negative mechanism of action. Indeed, quantitative mass spectrometry studies have shown that mutant K27M histone H3 comprises only 3–17% of total H3 protein in human DMG samples [29]. Early chromatin studies in these tumors uncovered a stark global loss of di- and tri-methylation at H3K27 (H3K27me2, me3) [29,52,53,54]. These epigenetic marks, which are associated with gene silencing, are catalyzed by the Polycomb repressive complex 2 (PRC2), a Polycomb group protein comprised of several subunits: the catalytic enhancer of zeste homolog 1 (EZH1) or EZH2, embryonic ectoderm development (EED), and suppressor of zeste 12 homolog (SUZ12). Subsequent evaluation of PRC2 components in the context of H3.3K27M mutation led to several hypothesized mechanisms underlying this H3K27me3 loss (Figure 2).

PRC2 enzymatic inhibition: In vitro studies showed that synthetic H3.3K27M peptide is able to impair PRC2 methyltransferase activity on both mutant and WT H3.3-containing nucleosomes in HEK293T cells [29]. Crosslinking assays demonstrated that the K27M peptide interacts with the EZH2 active site [29], further substantiated by the solution of the crystal structure of human PRC2 bound to H3.3K27M, which showed the mutant moiety in the active pocket of the SET domain of EZH2 [55]. In vitro histone methyltransferase assays demonstrated a 40–70% decrease in EZH2 activity in multiple cell lines with exogenous H3.3K27M, amplified to an 85% decrease in K27M mono-nucleosomes [52]. Additional in-depth biochemical analyses confirmed this inhibition of EZH2 methyltransferase activity as well as mapped the binding of EZH2 to the histone H3 tail [56]. Altogether, these studies suggest that H3.3K27M can directly inhibit the catalytic activity of the PRC2 methyltransferase complex.

PRC2 sequestration: Further analysis in these studies also demonstrated preferential binding of H3.3K27M to PRC2 components. Coimmunoprecipitation experiments in multiple cell types showed enrichment of EZH2 and SUZ12 at K27M mono-nucleosomes as compared with WT mono-nucleosomes [52]. H3.3K27M peptide bound to EZH2 16-fold more tightly than WT H3.3 in vitro [55], suggesting trapping and sequestration of PRC2 may play a role in the inhibitory effect of H3.3K27M. Studies in mouse embryonic stem cells found that EZH2 was redistributed and sequestered at poised enhancers (defined as chromatin regions marked by H3K27me3 and H3K4me1) that also contained H3.3K27M [57]. However, proteomic studies using quantitative mass spectrometry in Drosophila models failed to identify an enrichment of any PRC2 subunits with H3.3K27M-containing nucleosomes [58]. Furthermore, epigenomic profiling using ChIP-seq in DMG cells showed the PRC2 components EHZ2 and SUZ12 are largely excluded from K27M-containing chromatin [59], countering the sequestration theory.

Impaired H3K27me3 spread: A more recent study leveraging CRISPR-Cas9 technology to generate isogenic DMG cell lines also found that PRC2 recruitment to its high-affinity sites at unmethylated CpG islands is not altered in the presence of H3.3K27M [60], adding to the opposition of a simple sequestration model. Instead, they found that H3K27me3 deposition was restricted to narrow peaks surrounding these high affinity sites, with slightly broader deposition of H3K27me2 away from these sites. This impaired spread was reversible, as removal of the mutant K27M allele restored the wild type pattern of H3K27me2/me3 deposition. An independent investigation of H3.3K27M mutant and WT DMG cells confirmed this impaired spread of H3K27me3 from PRC2 high affinity sites and suggested enhanced allosteric inhibition of PRC2 by H3K27me3 in the presence of the K27M mutant as a possible mechanism [55,61]. These studies suggest more nuanced regulation of PRC2 localization in the presence of mutant K27M beyond direct inhibition of activity or direct binding and sequestration.

Additional epigenetic alterations: Such a nuanced model is supported by our broader understanding of chromatin modification mediated by H3.3K27M beyond loss of H3K27 trimethylation. For instance, several of the studies that initially described a global loss of H3K27me3 in K27M mutant cells also described a somewhat paradoxical gain of H3K27me3 at certain loci [52,53]. Further characterization of these loci revealed them to be strong Polycomb targets associated with CpG islands [27], as determined by density of H3K27me3 in H3WT cells and retention of H3K27me3 after treatment with EZH2 inhibitors. This is in keeping with the impaired spread of H3K27me3 from such sites in other studies [60]. As Polycomb target genes are intimately involved with development and differentiation, it has been suggested that these sites of retained or enhanced H3K27me3 may play just as important a role in tumorigenesis as the loss of H3K27me3. This theory is supported by impaired growth of DMG cells in the presence of an EZH2 inhibitor [27,59,60] as well as prolonged survival in a DMG preclinical model [27]. However, these findings were not replicated by other groups [22,62], and the role of H3.3K27M mutant status for this growth inhibitory effect remains unclear [27,60]. H3.3K27M also impacts activating chromatin marks such as H3K27 acetylation (H3K27ac), which is enriched in K27M mutant cells [29,58,63]. H3K27ac is a marker of super-enhancers that is bound by bromodomain and extra terminal (BET) proteins such as BRD2 and BRD4 to facilitate transcription initiation and elongation. These BET proteins are also found to be enriched at K27M-containing nucleosomes [58,59], and shRNA-mediated loss of BRD4 extended survival in a DMG xenograft model [64]. 

Yet another layer of complexity is added by considering the temporal evolution of these epigenetic effects. The use of an inducible H3.3K27M model showed that PRC2 initially colocalizes with K27M but that this colocalization decreases with time [61]. Despite no longer being physically associated, the methyltransferase activity of PRC2 from K27M mutant cells remains impaired, suggesting that H3.3K27M may induce a lasting perturbation in PRC2 such as a conformational change. Additional work has shown that H3.3K27M decreases EZH2 automethylation [65], which preferentially impairs PRC2-mediated conversion of H3K27me2 to me3, potentially explaining the more potent impact on H3K27me3 than H3K27me2 [60].

Changes in gene expression: Given the striking global loss of H3K27me3, one might have expected an accompanying global increase in gene expression due to the loss of silencing. However, RNA sequencing studies have not borne this out. In fact, comparison of H3K27 WT and H3K27M tumors show relatively modest differences in gene expression [23,52,53,60]. Nevertheless, these selective changes are broadly consistent, with multiple groups able to reliably separate H3.3K27M tumors using unsupervised clustering of gene signatures [5,9,10,66]. There is some variation in the exact set of differentially expressed genes between studies, which may in part be due to the multiple different modalities used to generate these signatures (tumors versus cell lines, primary samples versus genetically engineered models, human versus mouse origin). However, a persistent theme is the relative upregulation of PRC2 target genes by H3.3K27M as assessed by gene set enrichment analysis. Genes involved in neural development and differentiation are also upregulated in the presence of H3.3K27M [24,26,60], such as the inhibitor of differentiation (ID) gene family. A potential mechanism underlying these changes is the derepression of so-called “poised” or “bivalent” promoters, marked by the repressive H3K27me3 as well as the activating H3K4me3, by the loss of H3K27 trimethylation [23,67], as PRC2 targets and neural developmental genes are highly enriched in the population of bivalent promoters. An H3.3K27M-dependent redistribution of EZH2 to analogous poised enhancers has also been described [57]. This release of bivalent promoters parallels a process seen during normal developmental transitions [68] and is consistent with the framing of pediatric gliomagenesis as a corruption of normal developmental pathways and systems. Other studies have highlighted an oncogenic role for downregulation of tumor suppressor genes in the H3.3K27M context. For instance, the cell cycle gene CDKN2A isoform p16/INK4A locus often retains H3K27me3 in K27M-mutant cells, associated with decreased expression and accelerated tumor formation [22,27], though this finding is variably present [59].

Growth and transformation: The integrated impact of H3.3K27M mutation on relevant cell biology has also been extensively explored. Studies using both mouse and human neural progenitor cells engineered to express H3.3K27M in the presence of concurrent oncogenic mutations have demonstrated enhanced soft agar colony forming ability in the presence of K27M [26,27], suggesting this mutation promotes self-renewal capacity. This is supported by increased serial clonogenicity in neural stem cells derived from an H3.3K27M-based genetically engineered mouse model [23]. H3.3K27M mutation also confers a modest proliferative advantage in neural stem cells [23,26]; this effect appears to be narrowly restricted by cellular context, as no proliferative advantage was seen in embryonic stem cells or astrocytes [26]. This enhanced self-renewal and proliferation is coupled with altered differentiation potential. As referenced above, multiple gene ontology analyses of differentially expressed genes in the H3.3K27M context are enriched for neural lineage differentiation gene sets [22,23,59,60,67]. Impaired differentiation in H3.3K27M tumors has also be substantiated at the single cell level, where an OPC-like subset comprises the majority of cells across multiple different patient tumors [69]. This single cell RNA sequencing-based study also found an increased proportion of dividing and undifferentiated cells in K27M mutant tumors as compared with other canonical subgroups (such as IDH-mutant tumors). Functional assays of differentiation capacity showed reduced ability for H3.3K27M cells to generate astrocytes and, to a lesser extent, oligodendrocytes [26]. Of note, many of the studies investigating self-renewal and differentiation capacity were carried out in the context of H3.3K27M in combination with its frequent concurrent PDGFRA activation and p53 loss, while addition of K27M alone did not recapitulate these features. However, a study directly evaluating the role of H3.3K27M in these processes via shRNA-mediated knockdown in DMG xenografts confirmed an intrinsic promotion of stemness, proliferation, and impaired differentiation [67].

The exceptional spatio-temporal specificity of mutant tumors suggests a narrow developmental window in which a relevant cell type is susceptible to transformation by this mutation [70]. Profiling of these tumors suggests they recapitulate some aspects of the normal developmental hierarchy in neural and glial progenitor cells [69]. Indeed, many of the preclinical models described below revealed remarkable restrictions in the precursor cell populations competent to drive H3.3K27M-mediated gliomagenesis [24,26]. While both neural stem/progenitor cells and early oligodendrocyte precursor cells have been capable of tumor formation in different experimental models, elegant work comparing the resultant epigenomic remodeling has revealed disparate chromatin patterning in these two contexts, with the active chromatin landscape of transformed OPCs more closely resembling that of DMG tumors [71]. The geography of these transformed cells also plays a role, as hindbrain precursor cells seem preferentially susceptible to transformation by H3.3K27M [23,72]. This geographic predilection is supported by dysregulation of H3K27 methylation in other pediatric hindbrain tumors such as subsets of medulloblastoma [73] and posterior fossa ependymoma [74,75,76].

### 3.2. H3.1K27M

While the K27M mutation most commonly occurs at the histone H3.3 variant locus H3F3A, a minority of DMG harbor this mutation at histone H3.1 loci [12,13,77]. Initial characterization of these tumors grouped them with their H3.3 counterparts, as both demonstrate global reductions in H3K27 trimethylation [29]. However, more focused evaluations of these specific histone variant mutations have begun to uncover subtle differences in the dysregulation mediated by the two isoforms. Transcriptional and methylation-based profiling can reliably distinguish between H3.3 and H3.1 mutant tumors [8,78], with H3.3K27M correlating with proneural and oligodendroglial signatures, while H3.1K27M correlated with mesenchymal and astrocytic signatures. It is difficult to distinguish putative unique impacts of oncohistone isoforms from residual signatures derived from potentially distinct cells-of-origin or divergent concomitant mutations in bulk studies. Indeed, the H3.3K27M partner mutation PDGFRA and the H3.1K27M partner mutation ACVR1 have established roles in oligodendrocytic and astrocytic development, respectively. Evaluation of H3.3K27M and H3.1K27M in isogenic early OPCs does substantiate disparate chromatin localization of the histone variants as well as distinct patterns of active enhancers and gene expression even in the absence of additional mutations [71].

### 3.3. H3.3G34R/V

The molecular mechanisms underlying H3.3G34R/V-mediated oncogenesis are relatively uncharted by comparison, though they are clearly fundamentally distinct. While H3G34 itself is not subject to posttranslational modification, H3.3G34R/V affects trimethylation at the neighboring H3K36, an activating mark associated with transcriptional elongation [79]. In contrast to the dominant effect of H3K27M, this alteration occurs only in cis at mutant-containing nucleosomes [29,53]. Initial in vitro methylation assays demonstrated decreased H3K36 trimethylation at H3.3G34 mutant nucleosomes, possibly via impaired binding of the H3K36 trimethyltransferase SETD2 [29,53]. Subsequent structural modeling suggested steric hindrance of SETD2 binding by the large side chains in arginine or valine substitutions as the underlying mechanism [80]. The putative oncogenic role of H3K36me3 loss is bolstered by the discovery of mutually exclusive SETD2 missense and truncating mutations in cortical pHGG [81]. However, chromatin landscape profiling studies have shown a modest increase in H3K6me3 at certain genes such as *MYCN*, particularly those enriched in variant histone H3.3 deposition [66,82]. In vitro binding assays have shown preferential binding and inhibition of the H3K36 demethylase KDM4 by H3.3G34R/V [82], suggesting a dynamic interplay between H3K36 methylating and demethylating enzymes across specific genomic loci. 

Gene ontology analysis of ChIP-seq datasets between H3.3G34V and H3.3WT glioma cell lines showed enrichment in forebrain and cortical development gene lists [66], though it is difficult to ascribe these differences to the direct effect of H3.3G34V alone as opposed to potentially confounding variables such as the age, location, cell-of-origin, and concurrent mutations of the tumors of origin. In addition to its role in transcriptional elongation, H3K36me3 is also implicated in DNA mismatch repair [83]. Accordingly, H3.3G34R/V has been shown to confer defective homologous recombination-mediated mismatch repair [84] and an associated mild hypermutator phenotype [80]. Finally, very recent work has shown that H3.3G34R is preferentially bound by the chromatin reader RACK7 in pHGG cells, which leads to suppressed MHC class II protein expression and vesicular transport [85], alluding to a possible role in impaired antitumor immunity. 

## 4. Development of Novel Therapies

**Figure 3:**
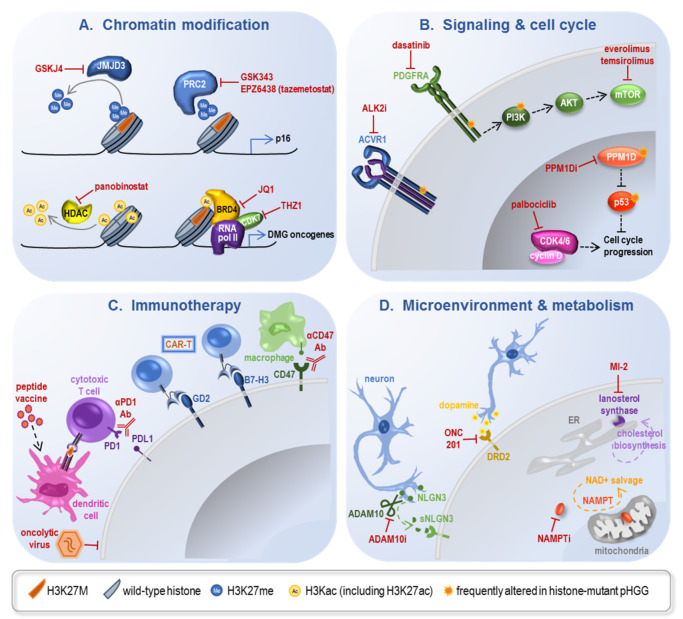
Strategies for molecular intervention in oncohistone-driven pHGG. Ac: acetylation; ER: endoplasmic reticulum; Me: methylation; RNA pol II: RNA polymerase II. See text for other abbreviations common to multiple tables and figures.

Steady progress in elucidating the molecular vulnerabilities of histone-mutant pHGG in conjunction with the development of biologically relevant mouse models has culminated in several preclinical studies of therapeutics tailored to the unique susceptibilities of these tumors [86], exploiting both the downstream consequences of oncohistone mutation as well as their obligate partner mutations (Figure 3). Several of these approaches now serve as the basis for ongoing clinical trials (Table 2). 

### 4.1. Reversal of Epigenetic Alterations 

Given the profound epigenetic ramifications of oncohistone mutation, it is perhaps not surprising that several approaches aim to reverse chromatin aberrations associated with these tumors.

H3K27me3 demethylase inhibition: As global loss of H3K27me3 is one of the molecular hallmarks of H3K27M pHGG, one of the first therapeutic studies evaluated pharmacologic inhibition of the K27 demethylase JMJD3 using GSKJ4 [87]. This led to restoration of H3K27me3 and improved survival in H3K27M orthotopic xenografts, with no effect in H3WT xenografts. 

EZH2 inhibition: Highlighting the nuanced regulation of H3K27me3 in these tumors, residual PRC2 methyltransferase activity remains required for effective tumor growth, as genetic EZH2 loss impaired H3.3K27M-engineered mouse NPC xenograft growth and EZH2 inhibition with two different compounds (GSK343, EPZ6438/tazemetostat) led to growth arrest in human pHGG cells [27,59], though there have been conflicting reports regarding this in vitro finding [22,62]. In vivo effects of small molecule EZH2 inhibition have not yet been decisively characterized.

BET inhibitors: Increased H3K27ac is another mainstay of H3K27M pHGG, and several strategies to either modify this aberrant acetylation or mitigate its effect have also proven fruitful. Pharmacologic inhibition of bromodomain proteins involved in H3K27ac recognition and subsequent transcription preferentially inhibited growth in H3K27M pHGG cells and extended survival in H3K27M PDX models [59]. Evaluation of a broader range of pHGG cell lines and BET inhibitors has confirmed the potential utility of this approach but highlighted the importance of potency and brain penetrance for further investigations [64]. 

CDK7 inhibition: Alternate means of attenuating the anomalous transcription present in H3K27M pHGG cells has also been explored via inhibition of CDK7, a cyclin-dependent kinase that phosphorylates RNA polymerase II to regulate consequent transcription. The CDK7 inhibitor THZ1 also potently inhibited H3K27M pHGG cell viability and led to a modest but significant improvement in xenograft survival [64]. In combination with the BET inhibitor studies, this suggests abrogation of the transcriptional consequences of altered H3K27ac as a promising strategy for H3K27M pHGG treatment.

HDAC inhibition: Interestingly, additional studies have intimated that increased H3K27ac may not contribute to pHGG pathogenesis and may in fact be exploitable as a vulnerability particular to H3K27M-mutant tumors. For instance, genome-wide mapping in H3K27M-mutant cells revealed pervasive increases in H3K27ac deposition throughout the genome that induced expression of repeat elements such as endogenous retroviral elements, with implications for antitumor immunity [63]. Polyacetylation at adjacent residues has also been shown to block the interaction of PRC2 with H3K27M and thus disrupt the PRC2 inhibition [56]. These findings potentially explain the therapeutic benefit of histone deacetylase (HDAC) inhibitors such as panobinostat. In a screen of dozens of promising agents using a panel of patient-derived DMG cell lines, HDAC inhibitors emerged as the leading category of interest, with panobinostat demonstrating marked impairment in cell viability as well as prolonged survival in PDX models [37]. This therapeutic effect has been substantiated by others, though the selective vulnerability of H3K27M-mutant tumors remains debated [63]. Importantly, pHGG cells that survived panobinostat treatment developed resistance to panobinostat re-challenge [37], underscoring the likely need for synergistic treatment strategies in this disease. 

Epigenetic Combination Therapies: Synergistic treatments have been validated in vitro with some of the epigenetic disrupters explored as monotherapy above, including BET inhibitors [37,64] and CDK7 inhibitors [64] combined with panobinostat. A more expansive in vitro followed by in vivo screen of the combinatorial druggable DMG landscape, with clinically relevant factors such as potency and blood–brain-barrier penetrance taken into account, revealed the proteasome inhibitor marizomib combined with panobinostat as the most promising pairing [88]. Additional in vivo studies have suggested inhibition of lysine demethylase [89,90], DNA methylation [63], AXL kinase [91], and PI3K [92] as promising combination strategies with panobinostat, several of which are now in clinical trials.

### 4.2. Blocking Oncogenic Signaling Pathways

Many of the canonical cooperating mutations in histone-mutant pHGG involve growth factor signaling or cell cycle regulation, emphasizing the importance of these pathways in promoting oncohistone-mediated gliomagenesis and prompting therapeutic trials of their blockade. 

ALK2 inhibition: Small molecule inhibition of ALK2 (ACVR1) with two different compounds demonstrated efficacy in ACVR1-mutant PDX models [93]. 

PPM1D inhibition: Inhibition of mutant PPM1D prolonged survival in a PDX model, and in vitro studies suggest synergy with ionizing radiation [94] and PARP inhibition [95] in PPM1D-mutant models. 

Growth factor signaling inhibitors: Growth factor signaling inhibitors have typically been explored in combination with additional treatments given concerns regarding the subclonal nature of relevant mutations as well as redundancy in the downstream signaling networks. PDGFRA signaling inhibition with dasatinib prolonged survival in an IUE-based H3.3K27M pHGG model, and combination with the mTOR inhibitor everolimus demonstrated synergy [33]. 

CDK4/6 inhibition: Blockade of cell cycle progression via CDK4/6 inhibition has also been pursued, in part given the role of upstream p16 downregulation in H3K27M pHGG. Palbociclib treatment improved survival in PDX models derived from untreated DMG biopsies [96], with in vitro evidence of synergy when combined with mTOR inhibition [97].

### 4.3. Immunotherapy

The largely immunosuppressive microenvironment of gliomas has hampered the application of immunotherapies that have shown efficacy in other cancer types. There are some suggestions that the microenvironment of pHGG is less immunosuppressive than that of its adult counterpart [98] and as such may be more amenable to immunomodulatory treatments [99]. 

Immunomodulatory and oncolytic viruses: An adenoviral-based TK/Flt3L immunostimulatory gene therapy approach showed efficacy in an ACVR1-mutant mouse model with histologic evidence of anti-tumor cytotoxic response [100]. Antitumor immunity has also been triggered using the oncolytic adenovirus Delta-24-RGD, with efficacy demonstrated in several immunodeficient and immunocompromised pHGG models [101]. Analogous to the concept of checkpoint inhibition, tumor immune evasion inhibition via disruption of CD47 binding to SIRPα prolonged survival in multiple pediatric brain tumor models including DMG [102].

Vaccines and adoptive cell therapies: Leveraging the tumor-specific nature of oncohistones, immune targeting of H3K27M has been pursued, with both peptide vaccine [103] and adoptive T cell receptor [104] paradigms showing preclinical promise. Other adoptive cell therapies such as chimeric antigen receptor (CAR) T cells targeting pHGG-associated antigens have demonstrated substantial efficacy in vivo, with anti-B7-H3 CAR-T [105] and anti-GD2 CAR-T [106] progressing to clinical trials. Of note, a minority of mice in the anti-GD2 CAR-T study developed on-target pontine inflammation that resulted in ventricular compression and hydrocephalus, a finding that raises caution for all immunotherapies targeting tumors with such a perilous neuroanatomical location.

### 4.4. Tumor Microenvironment and Metabolism

Emerging unconventional approaches to histone-mutant pHGG are galvanizing new areas of therapeutic research, including modifying input from neighboring neurons as well as capitalizing on particular metabolic susceptibilities in these tumors. 

ADAM10 inhibition: Neuronal activity can promote pHGG proliferation via release of postsynaptic neuroligin-3 cleaved by the metalloproteinase ADAM10, inhibition of which impairs PDX growth [107]. 

ONC201: Dopamine receptor D2 is highly expressed on some pHGG, and the dopamine receptor D2/D3 antagonist ONC201 has shown some suggestion of clinical activity in preliminary trials [108,109]. 

MI-2: Screening of a chemical library for inhibitors of H3K27M pHGG growth uncovered MI-2 as a lead candidate [26]. While this drug was initially characterized as a menin inhibitor, inhibition of lanosterol synthase and resultant disruption of cholesterol homeostasis appear to be its relevant mechanism of action [110]. 

Mitochondrial targeting: Tumor profiling indicated notable decrease in mitochondrial DNA quantity in pHGG, and shifting glucose metabolism from glycolysis to mitochondrial oxidation in combination with metformin to further target mitochondrial function and radiation to potentiate apoptosis was efficacious in in vivo models [111]. Recent work also suggests unique susceptibility of PPM1D-mutant pHGG to NAMPT inhibition in vivo [112], supporting the concept of pHGG-specific metabolic vulnerabilities.

## 5. Conclusions

Histone-mutant pHGG is an intricate and devastating disease with a paucity of clinically proven interventions. The unprecedented proliferation of relevant molecular studies in the past decade has deepened our understanding of its underlying oncogenic mechanisms and uncovered novel targets for therapy. Central to this ongoing effort has been the development of preclinical models in which to explore molecular mechanisms and screen potential therapeutics. Further work is needed to deconvolute the sometimes contradictory observations emerging from different models, which are likely due to the remarkable contextual specificity of these effects. Different cell types at different developmental ages from different neuroanatomic locations with different species of origin may show altered consequences of oncohistone mutation, highlighting the importance of developing model systems that accurately reflect the biology of the human disease. We are just beginning to unravel the mechanistic foundations of this idiosyncrasy, such as relative levels of H3K27M and PRC2 [61]. Incisive investigations leveraging our current complement of genomic editing, epigenomic profiling, and single cell sequencing tools is sure to further propel this accelerated progress, with meaningful therapeutic strategies on the horizon.

## Figures and Tables

**Table 1:** Experimental models of histone-mutant pHGG. DNp53: dominant negative p53; ESC: embryonic stem cell; GFAP: glial fibrillary acidic protein; [i]NPC: [induced] neural progenitor cell; OPC: oligodendrocyte precursor cell; Pax3: paired box gene 3. See text for other abbreviations common to multiple tables and figures.

Category	Technique	Genotype	Cell Type	Age	Phenotype	Reference
***Ex vivo* altered cells**	human iNPCs	H3.3K27M, *p53* loss, PDGFRA^D842V^	NPCs derived from human ESCs	neonatal (P6)	high grade glioma (grade III)	[26]
	mouse NPCs	H3.3K27M, PDGF-B	NPCs isolated from embryonic mouse forebrain	adolescent (6–10 weeks)	high grade glioma	[27]
		H3.3K27M, *p53* loss	NPCs isolated from embryonic mouse forebrain	adolescent (6–10 weeks)	no tumors	[27]
**GEMM**	RCAS/tv-a	PDGF-B, *p16* loss	nestin+ hindbrain NPCs	neonatal (P0–3)	high grade glioma	[28]
		H3.3K27M, *p53* loss, PDGF-B	nestin+ hindbrain NPCs	neonatal (P3–4)	high grade glioma	[22,29]
		H3.3K27M, *p53* loss	nestin+ hindbrain NPCs	neonatal (P3–4)	proliferating clusters	[29]
		H3.3K27M, *p53* loss, PDGF-B	Pax3+ hindbrain NPCs	neonatal (P3)	high grade glioma	[30]
		H3.1K27M, *p53* loss, PDGF-A, ACVR1^R206H^	nestin+ hindbrain NPCs	neonatal (P3–5)	high grade glioma	[31]
		H3.1K27M, *p53* loss, ACVR1^R206H^	nestin+ hindbrain NPCs	neonatal (P3–5)	proliferating clusters	[31]
		H3.1K27M, *p53* loss, *pten* loss ACVR1^R206H^	nestin+ hindbrain NPCs	neonatal (P3–5)	proliferating clusters	[31]
		H3.1K27M, *p53* loss, ACVR1^G328V^	nestin+ hindbrain NPCs	neonatal (P3–5)	proliferating clusters	[31]
		H3.1K27M, *p53* loss, *pten* loss ACVR1^G328V^	nestin+ hindbrain NPCs	neonatal (P3–5)	proliferating clusters	[31]
	*In utero* electroporation	H3.3K27M, *p53* loss ± PDGFRA, *ATRX* loss	forebrain or hindbrain periventricular NPCs	embryonic (E12.5–13.5)	high grade glioma	[24]
		H3.3K27M, *p53* loss	forebrain or hindbrain periventricular NPCs	neonatal (P0–2)	proliferating clusters	[24]
		H3.3K27M, *p53* loss, *ATRX* loss	forebrain or hindbrain periventricular NPCs	neonatal (P0–2)	proliferating clusters	[24]
		H3.3K27M, *p53* loss + PDGF-B, PDGFRA^WT^ or PDGFRA^D842V^	hindbrain periventricular NPCs	embryonic (E13.5)	high grade glioma	[32]
		H3.3K27M, DNp53, PDGFRA^D842V^	forebrain periventricular NPCs	embryonic (E13.5)	high grade glioma	[33]
		NRAS^V12^, *p53* loss, *ATRX* loss ± H3.3G34R	unknown	“postnatal”	high grade glioma	[25] SNO 2018
	Transgenic	H3.3K27M, *p53* loss, PDGFRA^V544ins^	nestin+ NPCs	neonatal (P0–1)	high grade glioma	[23]
		H3.3K27M, *p53* loss	nestin+ NPCs	neonatal (P0–1)	medulloblastoma, high grade glioma	[23]
		*p53* loss, PDGFRA^V544ins^	nestin+ NPCs	neonatal (P0–1)	high grade glioma	[23]
		H3.3K27M, *p53* loss	nestin+ NPCs	n/a	no tumors	[24]
		H3.3K27M, *p53* loss	GFAP+ NPCs	n/a	no tumors	[24]
		H3.1K27M, ACVR1^G328V^, PIK3CA^H1047R^	Olig2+ OPCs	n/a	high grade glioma	[34]
		H3.1K27M, ACVR1^G328V^	Olig2+ OPCs	n/a	proliferating clusters	[34]
**PDX**	orthotopic xenograft	H3.3WT, H3.3K27M and H3.1K27M tumors	n/a	neonatal (P2); adolescent (4–6 weeks)	high grade glioma	[35,36,37,38,39,40]; catalogued in [41]

**Table 2:** Ongoing clinical trials for histone-mutant pHGG. BMI1: B cell-specific Moloney murine leukemia virus integration site 1; CED: convection enhanced delivery; IDO: indoleamine 2,3-dioxygenase; IL12: interleukin 12; IV: intravenous; PO: oral; RT: radiotherapy; TMZ: temozolomide. See text for other abbreviations common to multiple tables and figures.

Category	Intervention	Administration	Trial Identifier	Tumor Eligibility	Phase
**HDACi & chromatin modifiers**	panobinostat	PO	NCT02717455 (PBTC-047)	nonprogressive; recurrent/refractory	I
	entinostat	PO	NCT02780804	recurrent/refractory	I
	valproic acid + RT/TMZ	PO	NCT03243461	newly diagnosed	III
	panobinostat nanoparticles (MTX110)	CED	NCT03566199 (PNOC015)	newly diagnosed	I/II
	vorinostat + temosirolimus ± RT	PO	NCT02420613	newly diagnosed; recurrent/refractory	I
	Fimepinostat *[dual HDACi/PI3Ki]*	PO	PNOC016	newly diagnosed; recurrent/refractory	I
	marizomib ± panobinostat	PO	NCT04341311	newly diagnosed	I/II
	PTC596 *[BMI1i]* + RT	PO	NCT03605550	newly diagnosed	Ib
**Immunotherapy**	cemiplimab (REGN2810) + RT	IV	NCT03690869 (PNOC013)	newly diagnosed; recurrent/refractory	I
	H3K27M vaccine + nivolumab	IV	NCT02960230 (PNOC007)	newly diagnosed	I
	pembrolizumab	IV	NCT02359565 (PBTC-045)	recurrent/refractory	I
	APX005M *[CD40 agonistic Ab]*	IV	NCT03389802 (PBTC-051)	newly diagnosed; recurrent/refractory	I
	indoximod + RT/TMZ	PO	NCT04049669	newly diagnosed	II
	IL12 adenovirus	intratumoral	NCT03330197	newly diagnosed	I/II
	B7-H3 CAR-T cells	intratumoral; intraventricular	NCT04185038	newly diagnosed; recurrent/refractory	I
	GD2 CAR-T cells	IV	NCT04099797	newly diagnosed	I
**Cytotoxic**	nanoliposomal irinotecan	CED	PNOC009	newly diagnosed	I/II
	gemcitabine	IV	NCT02992015	newly diagnosed	I
**Kinase Inhibitor**	abemaciclib ± RT	PO	NCT02644460	recurrent/refractory	I
	ribociclib + everolimus	PO	NCT03387020 (PBTC-050)	recurrent/refractory	I
	ribociclib + everolimus	PO	NCT03355794	newly diagnosed (s/p RT)	I
	palbociclib + TMZ + irinotecan	PO + IV	NCT03709680	recurrent/refractory	I
	dasatinib + everolimus	PO	NCT03352427	newly diagnosed; recurrent/refractory	II
	paxalisib (GDC-0084) *[dual PI3K/mTORi]*	PO	NCT03696355	newly diagnosed (s/p RT)	I
	Savolitinib	PO	NCT03598244 (PBTC-049)	recurrent/refractory	I
	Adavosertib (MK-1775) + RT	PO	NCT01922076	newly diagnosed	I
**Other**	ONC201 *[DRD2i]*	PO	NCT03416530	newly diagnosed; recurrent/refractory	I
	^124^I-8H9 (omburtamab)	CED	NCT01502917	nonprogressive	I
	INCB7839 *[ADAM10i]*	PO	NCT04295759 (PBTC-056)	recurrent/refractory	I

## Abbreviations

Ab	Antibody
ACVR1/ALK2	Activin a receptor, type 1
ADAM10	A disintegrin and metalloproteinase domain-containing protein 10
AKT	Protein kinase B
ATRX	Alpha-Thalassemia/Mental Retardation Syndrome, X-Linked
B7-H3	CD276 (target of 8H9 antibody)
BET	Bromodomain and extra-terminal motif
BRD4	Bromodomain-containing protein 4
CAR-T	Chimeric antigen receptor T cell
CDK	Cyclin-dependent kinase
DMG	Diffuse midline glioma, H3K27M-mutant
DRD2	Dopamine receptor D2
EZH2	Enhancer of zeste 2
Flt3L	FMS-like tyrosine kinase 3 ligand
GD2	Disialoganglioside GD2
GEMM	Genetically engineered mouse model
HDAC	Histone deacetylase
IUE	*In utero* electroporation
JMJD3/KDM6B	Jumonji domain-containing protein D3/lysine-specific demethylase 6B
MHC	Major histocompatibility complex
mTOR	Mechanistic target of rapamycin
NAD+	Nicotinamide adenine dinucleotide
NAMPT	Nicotinamide phosphoribosyltransferase
[s]NLGN3	[Soluble] neuroligin 3
NPC	Neural progenitor cell
OPC	Oligodendrocyte progenitor cell
PARP	Poly (ADP-ribose) polymerase
PD1/PDL1	Programmed death 1/programmed death ligand 1
PDGF-B	Platelet-derived growth factor subunit B
PDGFRA	Platelet-derived growth factor receptor A
PDX	Patient-derived xenograft
pHGG	Pediatric high-grade glioma
PI3K	Phosphatidylinositol 3-kinase
PIK3CA/PIK3R1	PI3K catalytic/regulatory subunits
PPM1D	Protein Phosphatase, Mg2+/Mn2+ Dependent 1D
PRC2	Polycomb repressive complex 2.
RCAS/t-va	Replication-competent avian sarcoma-leukosis virus long-terminal repeat with Splice acceptor/tumor virus A
SET	Su(var)3-9, Enhancer-of-Zeste and Trithorax
shRNA	Short hairpin RNA
SUZ12	Suppressor of zeste-12
TK	Thymidine kinase
